# Validation of the Brazilian version of the Hip Sports Activity Scale (HSAS) for patients with femoroacetabular impingement: a cross-sectional study

**DOI:** 10.1590/1516-3180.2021.0832.R1.11052022

**Published:** 2022-08-29

**Authors:** Letícia Nunes Carreras Del Castillo Mathias, Themis Moura Cardinot, Danúbia da Cunha de Sá-Caputo, Juliana Pessanha de Freitas, Mário Bernardo, Rafaela Maria de Paula Costa, Nathalia Sundin Palmeira de Oliveira, Liszt Palmeira de Oliveira

**Affiliations:** I MSc. Physiotherapist and Doctoral Student, Department of Medical Specialties, Postgraduate Programa in Medical Sciences, Universidade do Estado do Rio de Janeiro (UERJ), Rio de Janeiro (RJ), Brazil.; II PhD. Physical Educator and Professor, Department of Pharmaceutical Sciences, Instituto de Ciências Biológicas e da Saúde (ICBS), Universidade Federal Rural do Rio de Janeiro (UFRRJ), Seropédica (RJ), Brazil.; III PhD. Physiotherapist and Researcher, Department of Biophysics and Biometrics, Laboratório de Vibrações Mecânicas e Práticas Integrativas (LAVIMPI), Instituto de Biologia Roberto Alcântara Gomes, Policlínica Piquet Carneiro (PPC), Universidade do Estado do Rio de Janeiro (UERJ), Rio de Janeiro (RJ), Brazil.; IV BSc. Physiotherapist and Master's Student, Department of Biophysics and Biometrics, Laboratório de Vibrações Mecânicas e Práticas Integrativas (LAVIMPI), Instituto de Biologia Roberto Alcântara Gomes, Policlínica Piquet Carneiro (PPC), Universidade do Estado do Rio de Janeiro (UERJ), Rio de Janeiro (RJ), Brazil.; V PhD. Physiotherapist and Professor, Department of Biophysics and Biometrics, Laboratório de Vibrações Mecânicas e Práticas Integrativas (LAVIMPI), Instituto de Biologia Roberto Alcântara Gomes, Policlínica Piquet Carneiro (PPC), Universidade do Estado do Rio de Janeiro (UERJ), Rio de Janeiro (RJ), Brazil.; VI MSc. Physiotherapist, Department of Medical Specialties, Postgraduate Programa in Medical Sciences, Universidade do Estado do Rio de Janeiro (UERJ), Rio de Janeiro (RJ), Brazil.; VII MD. Orthopedist, Department of Medical Specialties, Faculdade de Ciências Médicas (FCM), Universidade do Estado do Rio de Janeiro (UERJ), Rio de Janeiro (RJ), Brazil.; VIII MD, PhD. Orthopedist and Professor, Department of Medical Specialties, Postgraduate Program in Medical Sciences, Universidade do Estado do Rio de Janeiro (UERJ), Rio de Janeiro (RJ), Brazil.

**Keywords:** Hip, Hip injuries, Surveys and questionnaires, Reproducibility of results, Sports, Exercise, Quality of life, Life quality, Outcome measurement, Validity, Reliability

## Abstract

**BACKGROUND:**

The Hip Sports Activity Scale (HSAS) is a hip-specific instrument for assessing the present levels of physical activity among patients with femoroacetabular impingement (FAI) syndrome. When evaluating treatment outcomes in patients with FAI syndrome, it is necessary to use joint-specific instruments and ones that can evaluate the levels of physical activity in these patients, such as the HSAS-Brazil.

**OBJECTIVE:**

To validate the HSAS-Brazil among a group of physically active patients after arthroscopic treatment of FAI syndrome.

**DESIGN AND SETTING:**

Cross-sectional research of quantitative and qualitative types using data obtained from July 2018 to October 2019.

**METHODS:**

A total of 58 patients of both genders diagnosed with FAI syndrome and who had undergone hip arthroscopy participated in this research. To establish reliability and validity, patients first answered the Brazilian versions of the 12-Item Short-Form Health Survey (SF-12), Nonarthritic Hip Score (NAHS), and HSAS; after a 48-hour interval, they answered the HSAS-Brazil again.

**RESULTS:**

For test-retest reliability, the interclass correlation was 0.908 (P < 0.001). The HSAS-Brazil correlated to the NAHS-Brazil (r = 0.63, P < 0.001), as well as the SF-12 (Physical Health) (r = 0.42, P = 0.001).

**CONCLUSION:**

The HSAS-Brazil was validated and proved to be a reliable and valid scale to assess sports activity levels in physically active patients with FAI syndrome after arthroscopic treatment.

## INTRODUCTION

Over the past decade, femoroacetabular impingement (FAI) syndrome has become a frequent source of hip pain in physically active patients with no radiological evidence of osteoarthritis. Initially reported by Ganz et al.,^1^ this event may result from two main configurations of an anatomical abnormality. The cam type of impingement, usually detected in young men, is triggered by an aberrant femoral head-neck junction such that the peripheral radius of the head moving into the acetabulum increases along with the range of motion of the hip. The pincer type of impingement, often seen in aged women, results from contact of the femoral head-neck junction on the acetabular rim as a consequence of acetabular over coverage. Most patients present associated forms of these two arrangements, categorized as mixed impingement.^2–4^

Sports and physical exercises that demand energetic and repeated flexion and internal hip rotation are often associated with symptomatic FAI syndrome. Throughout internal rotation in flexion, the anterior portion of the head-neck juncture approaches the anterosuperior portion of the acetabular rim. These trigger recurrent tension of the labrum and contiguous cartilage. FAI syndrome may also injure the cartilage in the hip joint and can be a subjacent reason for osteoarthritis (OA).^5–7^

Hip arthroscopy is an increasingly performed surgical procedure for youth and mature adults with hip-related pain or dysfunction. Indications for hip arthroscopy mainly consist of frequent pain and abnormal bony morphology related to FAI syndrome, labral tears, chondral imperfections, and ligamentum teres injuries.^8,9^

Youth and mature adults undergoing hip arthroscopy greatly desire to return to sports and physical activities.^8^ In 2013, Naal et al.^10^ published a hip joint-specific sports activity scale, the Hip Sports Activity Scale (HSAS), to define the present physical activity levels among patients with FAI syndrome. Since then, this scale has been extensively adopted in English-speaking countries. A translation and cultural adaptation of the HSAS into the Brazilian Portuguese language has already been produced.^11^

## OBJECTIVE

This study aimed to validate the Brazilian version of the HSAS (HSAS-Brazil) among a group of physically active patients after arthroscopic treatment of FAI syndrome.

## METHODS

### Type of study

This was a cross-sectional study of quantitative and qualitative nature using data obtained from July 2018 to October 2019.

### Ethical issues

The ethics committee of our institution approved the study (no. 998.832 – March 15, 2015), and all patients signed a free and informed consent statement. Dr. Florian D. Naal (first author of HSAS) was permitted to translate, cross-culturally adapt, and validate the HSAS for use in the Brazilian Portuguese language.

### Participants

A total of 58 patients of both genders participated in the study. They had received a medical diagnosis of FAI syndrome and had undergone hip arthroscopy. These patients were selected consecutively at a private healthcare clinic in Rio de Janeiro. Patients with proficient literacy (complete high school education) were included, regardless of gender or ethnicity. They all complained of hip pain and had either received a medical diagnosis of FAI syndrome or undergone hip arthroscopy. Patients presenting with visual or cognitive disorders that hindered reading and interpretation of the questionnaires or those who did not completely answer the questionnaires, either at the time of the first application or at the time of the second application, were excluded.

### Description of the Hip Sports Activity Scale (HSAS)

The HSAS determines the levels of physical activity among patients with FAI syndrome. The HSAS consists of nine distinct degrees of physical activity. It has nine topics graded from 0 to 8, with 0 for sedentary persons and 8 for high-performance athletes, without subscales.^10^

### Study protocol

The study protocol consisted of the following steps: requiring participants to fill out an identification and clinical assessment file with each patient's demographic and clinical characteristics and application of three self-administered questionnaires: the validated Brazilian version of the 12-Item Short-Form Health Survey (SF-12);^12^ validated Brazilian version of the Nonarthritic Hip Score (NAHS-Brazil);^13^ and HSAS-Brazil.^11^ The patients were asked to answer all three questionnaires (first application). Then, after a 48-hour interval, they were asked to answer only the HSAS-Brazil (second application). Finally, the second application of HSAS-Brazil was responded to using e-mail.

### Reliability

The reliability of the HSAS-Brazil was evaluated through intra-evaluator test-retest reliability. For this, it was necessary to apply the questionnaires to the same patient at two different times. These two applications were evaluated using the intraclass correlation coefficient (ICC), ascertaining whether the same effects were reproduced. ICC values less than 0.5 suggest poor reliability; values between 0.5 and 0.75, moderate reliability; values between 0.75 and 0.9, good reliability; and values greater than 0.90, excellent reliability.^14,15^ No inter-evaluator assessment was made due to the self-applicable scale characteristic, which does not demand any intermediation from the evaluator.

### Validity

The validity of the Brazilian version of HSAS was investigated through construct and content validity.^14,15^

### Construct and content validity

The validity of the HSAS-Brazil was evaluated by analyzing the strength of the correlation of its scores with those of the NAHS-Brazil and SF-12. The aim was to estimate whether the construct and content validity of the Brazilian version of HSAS was convergent with or divergent from those of the other two questionnaires. To assess construct convergence, correlations between scores were examined among the three questionnaires: the HSAS-Brazil, NAHS-Brazil (total score), and SF-12 (physical health subscale). To assess the divergence of construct, the correlation between the HSAS-Brazil score and SF-12 (mental health subscale) was examined. Spearman correlation coefficient was adopted to assess both the convergent and the divergent construct validity. This generates an indicator that can vary from –1 (perfect negative correlation) to +1 (perfect positive correlation), in which zero represents the lack of correlation between the studied variables.^14–16^

### Statistical analysis

Descriptive statistical analysis was used to delineate the survey population. The psychometric properties of reliability and validity needed for validating the Brazilian version of the HSAS questionnaire were evaluated statistically using the statistical package for the social sciences, SPSS (version 26, 2019; SPSS Inc, Chicago, Illinois, United States).

## RESULTS

### Patient characteristics

The participants selected were literate, but their level of schooling was only up to high school. Thirteen patients (22.4%) were female. The mean age of the patients was 39.4 years (range, 13 to 61 years) (Table 1).

**Table 1:** Patients’ characteristics and score values of the instruments used in this study

Parameter/Score	Patients sample
**Age (years)**	39.4 ± 12.3
**Female**	22.4%
**HSAS-Brazil**	2.4 ± 1.8
**NAHS-Brazil**	80.9 ± 21.4
**SF-12** (physical health subscale)	46.2 ± 10.3
**SF-12** (mental health subscale)	52.4 ± 9.3

HSAS-Brazil = Brazilian version of the Hip Sports Activity Scale; NAHS-Brazil = Brazilian version of the Nonarthritic Hip Score; SF-12 (physical health subscale) = Short-Form Physical Component Scale; SF-12 (mental health subscale) = Short-Form Mental Component Scale.

### Questionnaire results

Score values of each outcome measurement of the HSAS-Brazil, NAHS, and SF-12 questionnaires in the final testing are presented in Table 1.

### Intra-evaluator test-retest reliability

The ICC was 0.908 (P < 0.001), and the confidence interval (95% CI) ranged from 0.849 to 0.944.

### Construct and content validity

The HSAS-Brazil was moderately correlated with the NAHS-Brazil and weakly correlated with SF-12 physical and mental health subscales (Table 2). The HSAS-Brazil presented good content validity in patients with FAI syndrome.

**Table 2:** Correlations between the HSAS-Brazil and other instruments used in this study

Instruments	HSAS-Brazil	P value
**NAHS-Brazil**	0.63	< 0.001
**SF-12** (physical health subscale)	0.42	0.001
**SF-12** (mental health subscale)	0.30	0.021

HSAS-Brazil = Brazilian version of the Hip Sports Activity Scale; NAHS-Brazil = Brazilian version of the Nonarthritic Hip Score; SF-12 (physical health subscale) = Short-Form Physical Component Scale; SF-12 (mental health subscale) = Short-Form Mental Component Scale.

## DISCUSSION

The HSAS was initially created and validated for German-speaking patients with FAI syndrome and then cross-culturally adapted and validated for a North American English-speaking population.^10^ The HSAS was also translated and cross-culturally adapted into Swedish and Brazilian Portuguese languages.^11,17^ The Swedish version has already been validated. The current study showed that the Brazilian version of the HSAS is a reliable and valid scale to estimate sports activity levels in patients with FAI syndrome, comprising characteristics equivalent to those in the original version.^10,17^

For the validation of the HSAS-Brazil, a total of 58 patients (average age of 39.4 years) with FAI syndrome who had undergone hip arthroscopy were evaluated. In the English and the Swedish version, the numbers of patients studied were 29 and 30 (average age of 32.5 and 30.6 years, respectively). The mean age of the patients in the original study was similar to that of the patients in the present study.^10^

Intra-evaluator reliability is assessed when an evaluator applies the same assessment instrument on two different occasions to the same patient or when a patient responds to the same questionnaire alone at two different times. Intra-evaluator test-retest reliability estimates the fraction of the total variability of measurements. For example, the selected patients initially answered the Brazilian versions of three questionnaires: the SF-12,^12^ NAHS, and HSAS;^11,13^ after a 48-hour interval, they answered the HSAS-Brazil again. During this time, no new medication, therapy, or procedures that might have rapidly changed the patient's clinical state were introduced. The ICC in this study was 0.91, indicating excellent test-retest reliability, similar to the original research (0.94) and the Swedish version (0.93).^10,17^ The higher the test-retest reliability, the higher the instrument's internal reliability. Thus, the HSAS-Brazil has strong reliability for the complete questionnaire.

Construct validity represents the degree to which an instrument's scores are consistent with the hypothesis about expected internal relationships. Content validity represents the degree to which a measuring instrument can be considered a reasonable reflection of the construct to be measured. In this study, the convergent and divergent construct validities were assessed under the hypothesis that the physical health subscale score of the SF-12 and the total score of the NAHS-Brazil should show a moderate to high correlation between the instruments since the NAHS-Brazil has a domain on activity levels. A greater correlation between HSAS-Brazil and NAHS-Brazil would be expected because both are specific for hip evaluation. A low correlation would be expected between the HSAS-Brazil and SF-12 mental health subscale scores.^14,16^

For content validity (hip specificity), we analyzed the strength of the correlation between HSAS-Brazil and SF-12. We presumed moderate correlations (r = 0.50) with SF-12 (physical health sub-scale) (convergent validity). To support divergent validity, we presumed low or no correlations (r > 0.30) between the HSAS-Brazil and SF-12 (mental health subscale).^14,16^ Our results showed that there was no significant correlation between the HSAS-Brazil and SF-12 (mental health subscale) (r = 0.30), a low and statistically significant correlation between the HSAS-Brazil and SF-12 (physical health subscale) (r = 0.42), and a moderate and statistically significant correlation between the HSAS-Brazil and NAHS-Brazil (r = 0.63). Thus, HSAS-Brazil has good construct and content validity.

For the HSAS Swedish version, there was a high and statistically significant correlation between the HSAS and Tegner scores (r = 0.794), revealing good construct validity. No significant correlation was found between the HSAS and iHOT-12 or any of the Hip and Inguinal Outcome Score (HAGOS) subscales, unsurprisingly, aside from HAGOS “physical activity”, suggesting low content validity. The original study found a moderate to high and statistically significant correlation between HSAS and HOS.^10,17^

Patient-reported outcomes are appraised in epidemiological and clinical surveys using data reported by survey participants.^10,13,17,18^ There are two ways to obtain these patient-reported outcomes: the instruments can be completed by the survey participants (self-administered) or applied by an interviewer. Self-administered questionnaires benefit from not demanding a research team, as participants can complete the questionnaires in their own time at the survey site or at home by mail or web-based applications. Interviewer-administered questionnaires consume additional resources but provide extra control over measurement quality. Interviewers can apply the questionnaires in person or via telephone.^19,20^

Some recent studies investigated whether self-administered and interview-based questionnaires provided different results.^19,20^ Lozano et al.^19^ demonstrated that the format of administration has no significant repercussion on the measurements for assessing patients with intermittent claudication using the WIQ and EQ-5D instruments, provided that the patient can complete a self-applicable questionnaire. Puhan et al.^20^ administered the Medical Outcome Study – human immunodeficiency virus (HIV) questionnaire, European Quality of Life Scale (EuroQol), Feeling Thermometer, and Visual Function Questionnaire 25 every 6 months to volunteers engaged in the Longitudinal Study of Ocular Complications in acquired immunodeficiency syndrome (AIDS) using self- or interviewer-administration. A large print questionnaire was accessible for volunteers with visual deficiency. They concluded that administration templates did not significantly impact repeated measurements of patient-reported outcomes. For this reason, the present study used the self-administered format for the application of the SF-12,^12^ NAHS-Brazil,^13^ and HSAS-Brazil questionnaires.^11^

This study also has some limitations. First, the NAHS we used as a reference measure has been construct validated. Hence, the association between the HSAS and NAHS has to be considered supporting content validity (measuring the same content, i.e., hip) but not real construct validity. Another limitation is the absence of responsiveness data for the NAHS-Brazil, which was not evaluated.

When assessing treatment outcomes in patients with FAI syndrome, it is necessary to use not only joint-specific instruments, including the NAHS-Brazil or HOS-Brazil,^13,18^ but also instruments that can evaluate the levels of physical activity in these patients, especially the HSAS-Brazil.

The HSAS-Brazil scale is available in Annex 1.

**Annex 1:** The Brazilian version of the Hip Sports Activity Scale, HSAS-Brazil (Escala de Atividade Esportiva do Quadril, HSAS-Brasil).

Escala de Atividade Esportiva do Quadril (HSAS-Brasil)
**Por favor, marque na lista a seguir o mais alto nível de atividade esportiva ou recreacional atual que você consegue realizar.**
**8. Esportes de Competição** *(nível elite)*
Futebol, Hóquei, Futebol americano/Rugby, Artes marciais, Tênis, Atletismo, Esportes de quadra*, Vôlei de praia, Beisebol/Softbol.
**7. Esportes de Competição** *(nível elite)*
Surfe, Wakeboard.
**Esportes de Competição** *(ligas menores/estudantil)*
Futebol, Hóquei, Futebol americano/Rugby, Artes marciais, Tênis, Atletismo, Esportes de quadra*, Vôlei de praia, Beisebol/Softbol.
**6. Esportes de Competição** *(nível elite)*
Golfe, Ciclismo, Mountain bike, Natação, Remo, Hipismo.
**Esportes de Competição** *(ligas menores/estudantil)*
Surfe, Wakeboard.
**5. Esportes de Competição** *(ligas menores/estudantil)*
Golfe, Ciclismo, Mountain bike, Natação, Remo, Hipismo.
**Esportes Recreativos**
Futebol, Hóquei, Futebol americano/Rugby, Artes marciais, Tênis, Atletismo, Vôlei de praia.
**4. Esportes Recreativos**
Tênis, Surfe, Wakeboard, Esportes de quadra*, Beisebol/Softbol.
**3. Esportes Recreativos**
Ginástica aeróbica, Corrida, Musculação para membros inferiores, Hipismo.
**2. Esportes Recreativos**
Golfe, Ciclismo, Mountain bike, Natação, Remo, Dança, Patinação.
**1. Esportes Recreativos**
Natação, Andar de bicicleta, Caminhada em trilhas, Caminhada em alta velocidade.
**0. Nenhum Esporte Recreativo ou de Competição**
*Esportes de Quadra: Basquete, Squash, Handebol, Vôlei.
**Por favor, indique seu esporte preferido:** ________________________________________.

## CONCLUSION

The Brazilian version of the HSAS was validated and proved reliable for assessing sports activity levels in physically active patients after arthroscopic treatment of FAI syndrome. Therefore, the HSAS-Brazil can be a beneficial instrument for clinicians and researchers for detailed assessment of patients with FAI syndrome who practice sports and better compare distinct therapies or patient cohorts in terms of sports levels as a prognostic factor.
